# Association of maternal and infant inflammation with neurodevelopment in HIV-exposed uninfected children in a South African birth cohort

**DOI:** 10.1016/j.bbi.2020.08.021

**Published:** 2021-01

**Authors:** Tatum Sevenoaks, Catherine J. Wedderburn, Kirsten A. Donald, Whitney Barnett, Heather J. Zar, Dan J. Stein, Petrus J.W. Naudé

**Affiliations:** a Department of Psychiatry and Mental Health, University of Cape Town, South Africa; b Department of Paediatrics and Child Health, Red Cross War Memorial Children's Hospital, University of Cape Town, South Africa; c Neuroscience Institute, University of Cape Town, South Africa; d Department of Clinical Research, London School of Hygiene & Tropical Medicine, London, UK; e MRC Unit on Child and Adolescent Health, University of Cape Town, South Africa; f SU/UCT MRC Unit on Risk and Resilience in Mental Disorders, University of Cape Town, South Africa

**Keywords:** HIV exposed uninfected (HEU), Inflammation, Neurocognitive development, HIV infection, Birth cohort

## Abstract

HIV-exposed uninfected (HEU) children may have altered immune regulation and poorer neurodevelopment outcomes compared to their HIV-unexposed (HU) counterparts. However, studies investigating the association of maternal and infant inflammation with neurodevelopment in HEU children are limited and longitudinal data are lacking. This study investigated serum inflammatory markers in women living with HIV *vs*. HIV-uninfected women during pregnancy and in their children, as well as associations with neurodevelopmental outcomes at two years of age in an African birth cohort study. A sub-group of mother–child dyads from the Drakenstein Child Health Study had serum inflammatory markers measured at ≈26 week’s gestation (n = 77 HIV-infected mothers; n = 190 HIV-uninfected mothers), at 6–10 weeks (n = 63 HEU infants and n = 159 HU infants) and at 24–28 months (n = 77 HEU children and n = 190 HU children). Serum inflammatory markers [granulocyte–macrophage colony-stimulating factor (GM-CSF), interferon-γ (IFN-γ), interleukin IL-1β, IL-2, IL-4, IL-5, IL-6, IL-7, IL-8, IL-10, IL-12p70, IL-13, tumor necrosis factor-α (TNF-α), neutrophil gelatinase-associated lipocalin (NGAL) and metalloproteinase-9 (MMP-9)] were analyzed with a multiplex bead array and ELISA assays. The Bayley Scales of Infant and Toddler Development, third edition, was used to assess neurodevelopment at 24–28 months. After correcting for multiple comparisons, HIV infection during pregnancy was associated with lower serum levels of inflammatory markers in mothers at 26 weeks gestation (GM-CSF and MMP-9, *p < 0.05*) and HEU children at 6–10 weeks (IFN-γ and IL-1β, *p < 0.01*), and at 24–28 months (IFN-γ, IL-1β, IL-2 and IL-4, *p < 0.05*) compared to HIV-uninfected mothers and HU children. In HEU infants at 6–10 weeks, inflammatory markers (GM-CSF, IFN-γ, IL-10, IL-12p70, IL-1β, IL-2, IL-4, IL-6 and NGAL, *all p < 0.05*) were associated with poorer motor function at two years of age. This is the first study to evaluate the associations of follow-up immune markers in HEU children with neurodevelopment. These findings suggest that maternal HIV infection is associated with immune dysregulation in mothers and their children through two years of age. An altered immune system in HEU infants is associated with poorer follow-up motor neurodevelopment. These data highlight the important role of the immune system in early neurodevelopment and provide a foundation for future research.

## Highlights

• Inflammatory markers were lower in mothers living with HIV.
• Inflammatory markers were lower in HIV-exposed uninfected infants and children.
• Early life immune markers predicted impaired motor function at 2 years of age.

## 1 Introduction

Maternal Human Immunodeficiency Virus (HIV) infection affects maternal physiology and may have far-reaching consequences for the development of the exposed fetus and child. With wider access to antiretroviral therapy (ART) to prevent transmission of HIV from mother to child, the number of HIV-exposed uninfected (HEU) children is increasing. This is particularly relevant in South Africa, where the mother-to-child transmission rate dropped to < 5% in 2018, resulting in a current HEU child population of 3 500 000 (UNAIDS, 2019). However, HEU children are at increased risk of morbidity and mortality (Ajibola et al., 2018, Brennan et al., 2016, Evans et al., 2016), and impaired neurodevelopment (McHenry et al., 2018, Wedderburn et al., 2019b).

Several mechanisms have been proposed to explain the adverse outcomes in HEU children including; maternal and child immune dysregulation, ART toxicity, higher exposure to infectious diseases and socio-environmental factors (Wedderburn et al., 2019a). The impact of HIV infection on the adaptive immune system has been well described in adults (Mohan et al., 2014). However, few studies have examined maternal immunity in pregnancy in the context of HIV infection. Previous reports suggest that HIV impacts the maternal immune system by creating a pro-inflammatory environment, which is characterized predominantly by increased levels of certain cytokines, such as interleukin (IL-1, IL-6) and tumor necrosis factor-α (TNF-α) (Faye et al., 2007, Richardson and Weinberg, 2011, Sachdeva et al., 2008). Findings from studies have shown suppression of cytokine levels in pregnant women living with HIV (Maharaj et al., 2017, Moussa et al., 2001). Further, altered immune regulation may be present in HEU children (Abu-Raya et al., 2016a). The vulnerability of the immune system in HEU children is also suggested by the increase in morbidity and mortality in HEU infants (Afran et al., 2014). However, results on HEU children inflammatory profiles are inconsistent, with evidence for both increased levels (Dirajlal-Fargo et al., 2019, Miyamoto et al., 2017, Prendergast et al., 2017) as well as reduced levels of cytokines in HEU children (Borges-Almeida et al., 2011, Chougnet et al., 2000).

A recent *meta*-analysis found poorer cognitive and motor neurodevelopment in HEU children (McHenry et al., 2018). Additionally, we have found adverse language outcomes at 2 years in HEU infants in the Drakenstein Child Health Study (DCHS) in South Africa (Wedderburn et al., 2019b). Another study in Botswana showed that HEU children performed similarly on neurodevelopmental assessments at 2 years of age compared to HU children, although HEU children had increased adverse expressive language outcomes (Chaudhury et al., 2017). The biological mechanisms underlying neurodevelopmental impairments in HEU children remain largely unknown. Studies suggest that maternal immune activation and increased levels of certain inflammatory markers during pregnancy may contribute to poor fetal brain development (Bilbo and Schwarz, 2012, Hsiao and Patterson, 2012, Morelli et al., 2015, White et al., 2020). Due to the expanding population of HEU children and concerns about neurodevelopmental vulnerability in these children, the association between HIV, the immune system and neurodevelopment is a critical area of investigation.

Based on existing literature showing that inflammatory markers are increased in people living with HIV, we first hypothesized that pregnant women living with HIV would have an increased pro-inflammatory profile compared to HIV-uninfected pregnant women. Secondly, previous studies have found an association between increased levels of inflammatory markers with neurodevelopmental disorders, therefore we hypothesized that there would be an association between increased levels of inflammatory markers with poorer neurodevelopmental outcomes in children at 2 years of age.

The aims of this study were to investigate the impact of HIV on inflammatory profiles of pregnant women and their uninfected children through two years, and to assess whether these are associated with neurodevelopmental outcomes in the children at 2 years of age in the DCHS.

## 2 Methods

### 2.1 Study participants

A sub-sample of randomly selected mother–child pairs (n = 267) from the Drakenstein Child Health Study (DCHS), a large population-based birth cohort, as previously described, were included in this study (Stein et al., 2015, Zar et al., 2015). In brief, enrolment took place between 2012 and 2015 at two primary health care clinics, TC Newman and Mbekweni in Paarl, South Africa. All births occurred at Paarl Hospital. Pregnant women were enrolled if they were at least 18 years of age, planned to live in the region for at least 1 year and provided written informed consent (Stein et al., 2015, Zar et al., 2015).

This sub-study and the DCHS were approved by the Faculty of Health Sciences, Human Research Ethics Committee (HREC) of the University of Cape Town (HREC 401/2009 and HREC 648/2018).

### 2.2 Study procedures

#### 2.2.1 Variable measures and collection

Routine HIV testing of pregnant women was conducted to confirm HIV status in accordance with the Western Cape prevention of mother-to-child transmission (PMTCT) HIV guidelines (Pellowski et al., 2019). All HIV-exposed children received HIV testing as per local guidelines, and all HEU children in the sub-sample were confirmed negative. Detection of HIV was done at 6 weeks by PCR and at 9 months and 18 months by rapid antibody, PCR or ELISA (Zar et al., 2015).

Maternal CD4 cell count and viral load during pregnancy were measured, with the result closest to 26 weeks’ gestation taken to coincide with the immune variables. Viral load was categorized as below the detectable limit with < 40 copies/ml, detectable with 40–1000 copies/ml and unsuppressed with > 1000 copies/ml. All mothers living with HIV received antiretroviral therapy (ART) during pregnancy according to PMTCT guidelines at the time. Maternal ART initiation was categorized as ‘before pregnancy’ or ‘during pregnancy’. All HEU infants received prophylaxis (nevirapine alone or combined with zidovudine) from birth (Pellowski et al., 2019).

Blood serum samples were taken at 26 weeks gestation for mothers and at 6–10 weeks and 24–28 months for children as outlined in the DCHS (Zar et al., 2015).

Sociodemographic information was collected using an interviewer administered questionnaire adapted from items used in the South African Stress and Health Study. Mothers self-reported employment status, education level, asset ownership, household income and clinic during an antenatal study visit between 28- and 32-weeks gestation (Stein et al., 2015, Zar et al., 2015).

Upon delivery detailed birth data was obtained, including mode of delivery, gestational age, infant sex, head circumference, infant length and infant birth weight. Gestational age was calculated using the best estimated delivery date based on the last menstrual period, antenatal ultrasound, or the symphysis-fundal height. Prematurity was defined at <37 weeks gestation (Budree et al., 2017b). Maternal Body Mass Index (BMI) was determined at 6 weeks postpartum (Budree et al., 2017b). Self-reported information on child feeding practices was obtained at infant follow-up visits at 6–10 weeks, and 24–28 months of age. At 6–10 weeks infants were categorized as exclusively breastfed if mothers were still breastfeeding but neither solids nor formula had been introduced (Budree et al., 2017a, Wedderburn et al., 2019b, Zar et al., 2015).

Maternal alcohol use during pregnancy was assessed using the Alcohol, Smoking, and Substance Involvement Screening Test; mothers were classified with either moderate-severe alcohol exposure *vs.* un-exposed (Stein et al., 2015). Infants and children were classified as being alcohol-exposed *in utero* if their mother was classified with moderate-severe alcohol exposure. Smoke exposure was measured at 26 weeks gestation using urine cotinine levels that were determined using the IMMULITE 1000 nicotine metabolite kit (Vanker et al., 2016). Mothers were considered a non-smoker if cotinine levels were < 10 ng/ml, a passive smoker with levels between 10 and 500 ng/ml and an active smoker with levels > 500 ng/ml.

#### 2.2.2 Neurodevelopmental assessment

Neurodevelopment of the children at 24–28 months was assessed with the Bayley Scales of Infant and Toddler Development, third edition (BSID-III) assessment (Bayley, 2006, Donald et al., 2018). The BSID-III is a well-validated tool that assesses child cognitive, language and motor development from 1 to 42 months and is sensitive to developmental delay (Bayley, 2006). The BSID-III testing was performed by trained assessors that were blinded to maternal HIV status. Training was performed in accordance with the BSID manual by a pediatric neurodevelopmental specialist who periodically monitored the assessors throughout the testing period to validate standardized data collection across sites and ensure agreement between assessors on both administration and scoring. The assessors alternated between the TC Newman and Mbekweni clinics and assessed equal number of children at each site. Assessments were performed with prompts in the child’s preferred language. External scoring quality control checks were also performed centrally before data capture. For the current study standardized composite scores were used. Composite scores were generated for each cognitive, language and motor domain, with a mean of 100 and standard deviation of 15, using normative United States data. The use of these composite scores have been validated in the South African setting (Ballot et al., 2017, Rademeyer and Jacklin, 2013).

#### 2.2.3 Immune assays

Pro- and anti-inflammatory cytokines have been used as an indicator of immune regulation in the majority of existing studies with HEU children (Borges-Almeida et al., 2011, Chougnet et al., 2000, Dirajlal-Fargo et al., 2019, Miyamoto et al., 2017, Prendergast et al., 2017), pregnant women living with HIV (Faye et al., 2007, Maharaj et al., 2017, Moussa et al., 2001, Richardson and Weinberg, 2011, Sachdeva et al., 2008) and brain disorders in children (Miyamoto et al., 2017). Serum neutrophil gelatinase-associated lipocalin (NGAL) levels were shown to be associated with cognitive impairments and reduced brain volumes in people living with HIV (Williams et al., 2019, Williams et al., 2020). NGAL levels were increased in the neocortex of post-mortem brain tissues from a subset of people living with HIV that had HIV-associated neuropathology. NGAL levels correlated with viral load in the CSF and pro-viral DNA in the cortex (Ojeda-Juárez et al., 2020). The study by Ojeda-Juárez and colleagues further showed that NGAL may play an important function in neuronal damage and neuroinflammation in a HIVgp120 transgenic mouse model for HIV (Ojeda-Juárez et al., 2020). Decreased plasma MMP-9 levels were found in South African people living with HIV (Williams et al., 2019). Evidence from studies in animals show that optimal maternal and infant MMP-9 levels are crucial for early life brain developmental processes (Reinhard et al., 2015).

NGAL and MMP-9 concentrations were measured using commercially available ELISA kits (NGAL: DY1757, MMP-9: D911; R&D systems) in serum samples obtained from the mothers at 26 weeks gestation and the HEU children at 6–10 weeks and 24–28 months. Pro- and anti-inflammatory immune markers (GM-CSF, INF-γ, IL-1β, IL-2, IL-5, IL-6, IL-7, IL-8 and TNF-α, IL-4, IL-10, IL-12p70 and IL-13) were analyzed with a Milliplex® Luminex premix 13-plex kit (HSTCMAG28SPMX13; Merck) according to the manufacturer’s instructions. Plates were read on a Luminex system (Bio-Plex 200 System; Bio-Rad). All samples were assayed in duplicate.

### 2.3 Statistical analysis

All data were tested for normality. Because of the skewed distribution, all markers were natural logarithm (ln) transformed. This resulted in acceptable skewness and kurtosis of the data, which were used for further statistical analyses. Cytokine levels below the level of detection (GM-CSF (n = 9), INF-γ (n = 2), IL-1β (n = 33), IL-2 (n = 5), IL-5 (n = 13), IL-6 (n = 5), IL-4 (n = 5), IL-10 (n = 3), IL-12p70 (n = 3) and IL-13 (n = 17)) were replaced by values representing half of the lowest value on the standard curve. Variables with missing values were: smoking status (n = 8), alcohol use (n = 14), socioeconomic status (n = 5), BMI at 6 weeks (n = 51), breastfeeding at 6–10 weeks (n = 20), viral load (n = 26), CD4 + count (n = 19) and finally birth weight (n = 2). Data was tested for random missingness using Little’s Missing Completely at Random (MCAR) test that showed a non-significant coefficient, χ2 = 25.48 (df = 29; p = 0.653). The data was therefore considered either MCAR or missing at random (MAR) and imputed using multiple imputation approach by chained equations (MICE) to replace the missing values (Rubin and McHugh, 1987). Statistical analyses were performed on ten imputed datasets. The Benjamini-Hochberg procedure was used to control for the false discovery rate throughout the analyses due to multiple testing (McDonald, 2009).

First, unpaired *t*-tests were used to explore the differences of the inflammatory markers in mothers during pregnancy and children at 6–10 weeks and 24–28 months according to maternal HIV status. Significant correlations were subsequently used in linear regression analyses with the respective markers as dependent variables, maternal HIV status as predictor and adjusted for covariates. Pearson’s correlations were used to explore the associations between the inflammatory markers at each time point with neurodevelopment measures at 24–28 months of age. Multivariable regression models were then performed separately on significant findings with the respective inflammatory markers as predictors and neurodevelopment measure as dependent variable. Covariates were selected a priori based on their potential effects on either inflammatory markers or neurodevelopment. The following variables were added to the model: unadjusted; Model 1) maternal sociodemographic and lifestyle factors (adjusted for clinic, maternal smoking during pregnancy, maternal alcohol use during pregnancy, maternal socioeconomic status, maternal BMI at 6 weeks postpartum); model 2) infant health (adjusted for birth weight, prematurity, infant sex and exclusive breastfeeding (yes/no)); model 3) maternal HIV disease parameters (adjusted for maternal CD4+ during pregnancy, maternal viral load during pregnancy, maternal ART regimen during pregnancy and initiation of ART (before or during pregnancy)). All analyses were conducted using SPSS (version 25, IBM, USA). Group differences were considered statistically significant for all analyses where p-values were<0.05.

## 3 Results

### 3.1 Participants

Table 1 presents demographic data for mothers included in the study; sociodemographic characteristics were similar across comparison groups. There were significantly more women living with HIV from Mbekweni compared to TC Newman clinic *(p < 0.001)* as well as more mothers living with HIV with moderate-severe alcohol exposure compared to HIV-uninfected mothers (*p = 0.032*).

**Table 1:** Descriptive demographic characteristics for HIV-infected and HIV-uninfected mothers and neonatal measures at birth.

MOTHERS	HIV-infected	HIV-uninfected	P-value
N (%)	77 (28.8)	190 (71.2)	
Age, mean (SD)	29 (5.43)	26 (5.87)	0.377
Site, N (%)			**<0.001**
*Mbekweni*	70/77 (90.9)	70/190 (36.8)	
*TC Newman*	7/77 (9.1)	120/190 (63.2)	
Education, N (%)			0.289
*Primary*	8/77 (10.4)	14/190 (7.4)	
*Some secondary*	50/77 (64.9)	108/190 (56.8)	
*Secondary*	17/77 (22.1)	56/190 (29.5)	
*Tertiary*	2/77 (2.6)	12/190 (6.3)	
Married, N (%)			0.933
*Married/cohabitating*	29/77 (37.7)	75/190 (39.5)	
*Other*	48/77 (62.3)	115/190 (60.5)	
Body Mass Index (BMI), mean (SD)	28.81 (6.60)	26.23 (6.06)	0.551
Smoking (urine cotinine levels ng/ml), N (%)			0.235
*Non-smoker (<10 ng/ml)*	21/74 (28.4)	37/185 (20.0)	
*Passive smoker (10*–*500 ng/ml)*	31/74 (41.9)	76/185 (41.1)	
*Active smoker (>500 ng/ml)*	22/74 (29.7)	72/185 (38.5)	
Alcohol exposure, N (%)			**0.032**
*Unexposed*	61/68 (89.7)	179/185 (96.8)	
*Moderate-severe exposure*	7/68 (10.3)	6/185 (3.2)	
Illness during pregnancy, N (%)	11/77 (14.2)	17/190 (8.9)	0.213
Maternal CD4 + count in pregnancy, median (range)	453.50 (339.25–628.00)	–	–
Viral load during pregnancy, N (%)			
*Below detectable limit (<40 copies/mL)*	40/51 (78.4)	–	–
*Detectable (≥40-1000 copies/mL)*	5/51 (9.8)	–	–
*Unsuppressed (>1000 copies/mL)*	6/51 (11.8)	–	–
Antiretroviral regimen during pregnancy, N (%)			
*Prevention of mother-to-child transmission*			
*prophylaxis (zidovudine)*	11/77 (14.3)	–	–
*First-line triple therapy*	64/77 (83.1)	–	–
*Second-line or third-line therapy*	2/77 (2.60)	–	–
Initiation of antiretroviral treatment, N (%)			
*Before pregnancy*	32/77 (41.6)	–	–
*During pregnancy*	45/77 (58.4)	–	–
Social-economic status (SES), N (%)			0.475
*Lowest SES*	25/77 (32.5)	43/185 (23.2)	
*Low-moderate SES*	17/77 (22.1)	49/185 (26.5)	
*Moderate-high SES*	20/77 (26.0)	55/185 (29.7)	
*High SES*	15/77 (19.5)	38/185 (20.5)	
Prematurity (<37 weeks), N (%)	8/77 (10.4)	26/188 (13.8)	0.464
Birth weight, mean (SD)	3.02 (0.52)	3.02 (0.59)	0.202
Delivery Method, N (%)			0.052
*Vaginal*	48/77 (69.6)	151/181 (83.4)	
*Elective Cesarean*	6/77 (8.7)	9/181 (5.0)	
*Emergency Cesarean*	15/77 (21.7)	21/181 (11.6)	

SD: standard deviation, N: numbers.

Table 2 presents characteristics of HU and HEU children at 6–10 weeks and at 24–28 months. Both HEU and HU groups had similar growth indices at 6–10 weeks and 24–28 months. More HU infants were exclusively breastfed at 6–10 weeks compared to HEU infants (*p = 0.032*). However, overall low rates of exclusive breastfeeding at 6–10 weeks occurred (45.5% in HEU and 60.8% in HU group), similar to findings across the cohort (Budree et al., 2017a). HEU children of this sub-study did not present with significant impaired neurodevelopmental scores compared to HU children.

**Table 2:** Descriptive characteristics for HEU and HU children at 6–10 weeks and 24–28 months.

INFANTS 6 WEEKS	HEU	HU	P-value
N (%)	63 (71.6)	159 (28.4)	
Age (weeks), mean (SD)	8.1 (1.5)	7.9 (1.6)	0.277
Baby sex (Female) N (%)	37 (59.7)	89 (57.1)	0.723
Weight (Kg), mean (SD)	4.91 (0.70)	4.83 (0.79)	0.505
Length (cm), mean (SD)	54.75 (2.66)	54.67 (2.90)	0.370
Head circumference (cm), mean (SD)	38.86 (1.54)	38.61 (1.81)	0.197
Exclusive breast feeding, N (%)	30 (45.5)	110 (60.8)	**0.032**
Cotrimoxazole use, N (%)	50 (80.6)	–	**–**

CHILDREN 24 MONTHS			

N (%)	77 (28.8)	190 (71.2)	
Age (months), mean (SD)	27.3 (4.0)	27.2 (3.5)	0.331
Baby sex (Female) N (%)	47 (61.0)	113 (59.5)	0.813
Weight (Kg), mean (SD)	11.98 (1.88)	11.61 (1.63)	0.249
Length (cm), mean (SD)	83.40 (3.65)	83.73 (3.54)	0.889
Head circumference (cm), mean (SD)	48.43 (2.15)	47.96 (1.89)	0.858
Cognitive, mean (SD)	85.62 (8.68)	86.64 (9.05)	0.641
Language, mean (SD)	82.42 (10.26)	85.20 (12.55)	0.118
Motor, mean (SD)	94.38 (11.83)	94.57 (13.52)	0.854

Abbreviations: HEU, HIV-exposed uninfected; HU, HIV-unexposed; SD, Standard deviation; N, numbers; Kg, Kilograms; cm, centimeters.

### 3.2 Inflammatory markers according to maternal HIV status

**Fig. 1:**
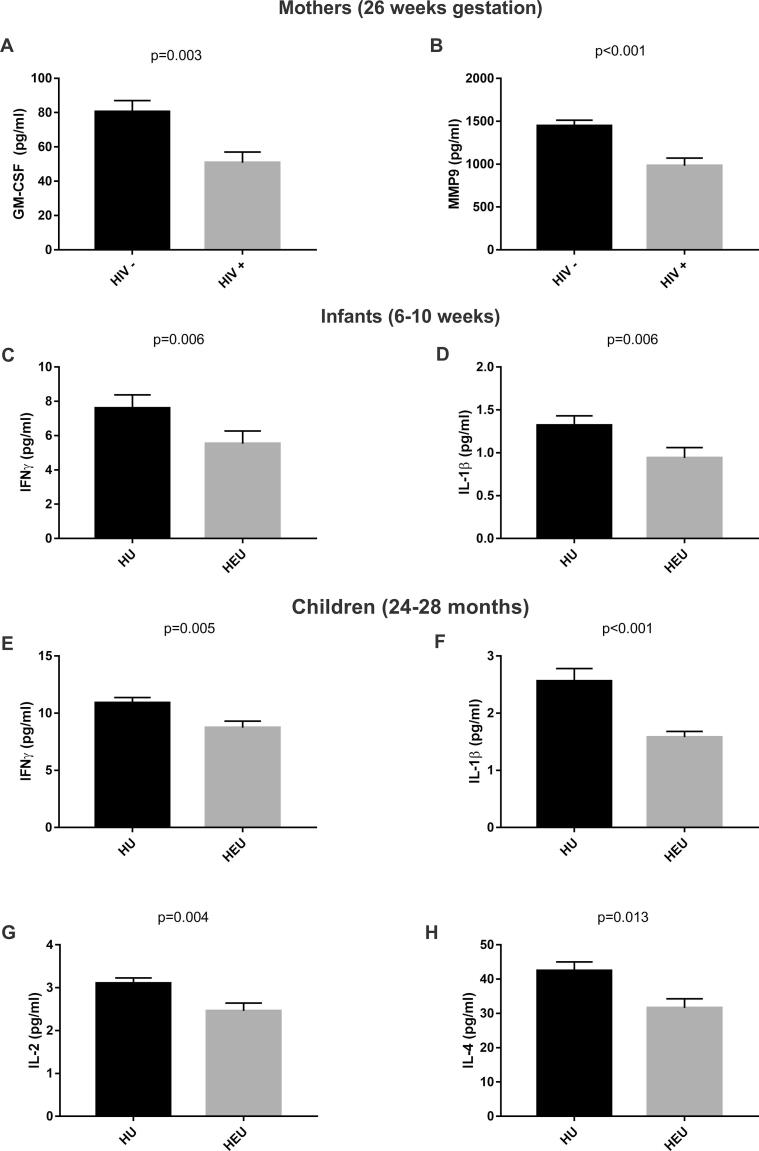
Comparisons of serum levels of all inflammatory markers that were significantly different in mothers living with HIV and their HEU children compared to HIV uninfected mothers and HU children.

Serum levels of the inflammatory markers GM-CSF *(p = 0.003) (*Fig. 1*A)* and MMP-9 *(p < 0.001) (*Fig. 1*B)* were significantly lower in mothers living with HIV compared to HIV-uninfected mothers at 26 weeks gestation. The inflammatory markers IL-1β (*p = 0.021*) and IL-4 (*p = 0.019*) were also lower in mothers living with HIV (*p < 0.05 uncorrected*), however this was not significant after multiple comparison correction.

In infants at 6–10 weeks serum levels of the inflammatory markers IFN-γ (*p = 0.006*) *(*Fig. 1*C)* and IL-1β (*p = 0.006*) *(*Fig. 1*D)* were decreased in HEU compared to HU infants. The inflammatory markers IL-12p70 and IL-4 were lower in HEU infants (*p < 0.05 uncorrected*) but this was not significant after multiple comparison correction.

In children at 24–28 months serum levels of inflammatory markers IFN-γ (*p = 0.005*) *(*Fig. 1*E)*, IL-1β (*p < 0.001*) *(*Fig. 1*F)*, IL-2 (*p = 0.004*) *(*Fig. 1*G)* and IL-4 (*p = 0.013*) *(*Fig. 1*H)* were all significantly lower in HEU compared with HU children after multiple comparison correction.

Fig. 1 indicates all inflammatory markers that were significantly reduced in mothers living with HIV and their HEU children compared to HIV-uninfected mothers and their HU children. The natural log (ln) transformed values for all the inflammatory markers for the mothers living with HIV and HIV-uninfected mothers and their children are shown in the supplementary file *(*supplementary Tables 1-3*)*.

Multivariable linear regression analyses were performed for inflammatory markers according to maternal HIV status *(*Table 3*)*. In adjusted analyses, GM-CSF *(p = 0.016)* and MMP-9 *(p = 0.034)* was significantly lower in mothers living with HIV. At 6–10 weeks, IFN-γ *(p = 0.001)* was significantly lower in HEU compared to HU infants; IL-1β *(p = 0.120)* was not significant after controlling for all covariates. By 24–28 months, IFN-γ *(p = 0.019)* IL-1β *(p = 0.001)*, IL-2 *(p = 0.035)* and IL-4 *(p = 0.017)* levels were significantly lower in HEU children after controlling for covariates.

**Table 3:** Multivariable linear regression analyses of inflammatory markers in mothers, infants (6–10 weeks) and children (24–28 months).

	B (SE)	β	P-value
Mothers*			
GM-CSF	−0.060 (0.03)	−0.122	0.016
MMP-9	−0.076 (0.04)	−0.124	0.034

Infants 6–10 weeks#			
IFN-γ	−0.088 (0.03)	−0.194	0.001
IL-1β	−0.048 (0.03)	−0.098	0.120

Children 24–28 months†			
IFN-γ	−0.097 (0.04)	−0.168	0.019
IL-1β	−0.110 (0.03)	−0.182	0.001
IL-2	−0.075 (0.04)	−0.116	0.035
IL-4	−0.060 (0.03)	−0.128	0.017

Multivariable regressions were performed separately for each inflammatory marker with significant differences after correcting for multiple comparisons, according to maternal HIV status as depicted in Fig. 1.

Abbreviations: GM-CSF, Granulocyte-macrophage colony-stimulating factor; MMP-9 metalloproteinase-9; IFN-γ, interferon-γ; IL, interleukin

* covariates: clinic, smoking, alcohol exposure, age, socioeconomic status and body mass index (BMI) at 6 weeks postpartum.

# **covariates**: clinic, maternal smoking during pregnancy maternal alcohol exposure during pregnancy, maternal socioeconomic status and maternal BMI at 6 weeks postpartum, prematurity, birth weight, exclusive breastfeeding at 6–10 weeks and infant sex.

† **covariates**: clinic, maternal smoking during pregnancy, maternal alcohol exposure during pregnancy, prematurity, maternal socioeconomic status and maternal BMI at 6 weeks postpartum, birth weight and child sex.

### 3.3 Association of inflammatory markers with neurodevelopment

No inflammatory markers in mothers living with HIV were significantly associated with neurodevelopmental measures in HEU children after correction for multiple comparisons. However, markers of inflammation in mothers living with HIV [IFN-γ (*r = -0.295, p = 0.01*), IL-10 (*r = -0.253, p = 0.036*), IL-12p70 (*r = -0.262, p = 0.030)* and IL-7 (*r = -0.246, p = 0.041*)] were associated with lower composite scores for language in HEU children (24–28 months) on initial analysis; but were not significant after multiple comparison correction. TNF-α (*r = -0.288, p = 0.014*) was also associated initially with lower cognitive scores in HEU children (24–28 months) prior to multiple comparison correction *(*Table 4*)*.

**Table 4:** Correlation between inflammatory markers in mothers living with HIV and HEU children at 6–10 weeks and 24–28 months with neurodevelopment outcomes at 24–28 months.

HIV-infected Mothers																
		GM-CSF	IFN-γ	IL-10	IL-12p70	IL-13	IL-1β	IL-2	IL-4	IL-5	IL-6	IL-7	IL-8	TNF-α	NGAL	MMP9
Cognitive	*r*	0.085	-0.131	-0.069	-0.045	-0.051	-0.023	-0.086	0.049	-0.128	-0.144	-0.226	-0.071	-0.288	-0.016	-0.122
	P-value	0.477	0.272	0.566	0.705	0.669	0.850	0.475	0.684	0.283	0.227	0.057	0.556	**0.014**	0.892	0.307
Language	*r*	-0.142	**-0.295**	**-0.253**	**-0.262**	-0.174	-0.223	-0.232	-0.208	-0.211	-0.204	**-0.246**	-0.128	-0.206	0.081	0.045
	P-value	0.245	**0.014**	**0.036**	**0.030**	0.153	0.066	0.056	0.086	0.081	0.093	**0.041**	0.295	0.089	0.509	0.711
Motor	*r*	0.031	-0.166	-0.147	-0.164	-0.082	-0.133	-0.085	-0.123	-0.134	0.012	-0.109	-0.029	-0.138	-0.036	-0.068
	P-value	0.807	0.182	0.240	0.189	0.511	0.288	0.496	0.325	0.283	0.923	0.383	0.815	0.269	0.775	0.589

HEU Infants (6–10 weeks)																
		GM-CSF	IFN-γ	IL-10	IL-12p70	IL-13	IL-1β	IL-2	IL-4	IL-5	IL-6	IL-7	IL-8	TNF-α	NGAL	MMP9
Cognitive	*r*	0.005	-0.178	-0.136	-0.089	-0.091	-0.207	-0.126	-0.164	-0.010	-0.181	-0.238	-0.225	-0.178	-0.153	-0.186
	P-value	0.971	0.178	0.305	0.501	0.492	0.116	0.343	0.214	0.938	0.171	0.070	0.086	0.177	0.251	0.162
Language	*r*	0.081	-0.106	-0.116	-0.055	-0.025	-0.199	0.007	-0.182	-0.070	-0.165	-0.061	-0.221	-0.152	-0.169	**-0.305**
	P-value	0.548	0.430	0.390	0.684	0.855	0.138	0.958	0.177	0.607	0.220	0.654	0.098	0.260	0.214	**0.022**
Motor	*r*	**-0.309^#^**	**-0.339^#^**	**-0.451^#^**	**-0.379^#^**	-0.243	**-0.491^#^**	**-0.308^#^**	**-0.418^#^**	-0.237	**-0.335^#^**	-0.239	-0.003	-0.071	**-0.383^#^**	**-0.289**
	p-value	**0.022**	**0.011**	**0.001**	**0.004**	0.074	**0.000**	**0.022**	**0.002**	0.082	**0.012**	0.079	0.981	0.607	**0.004**	**0.034**

HEU Children (24–28 months)																
		GM-CSF	IFN-γ	IL-10	IL-12p70	IL-13	IL-1β	IL-2	IL-4	IL-5	IL-6	IL-7	IL-8	TNF-α	NGAL	MMP9
Cognitive	*r*	-0.032	-0.191	**-0.303**	-0.166	0.018	-0.148	-0.148	-0.118	0.013	-0.106	-0.177	0.010	-0.054	0.207	-0.007
	P-value	0.793	0.111	**0.010**	0.166	0.884	0.217	0.219	0.329	0.916	0.381	0.139	0.933	0.654	0.081	0.956
Language	*r*	-0.234	**-0.307**	**-0.319**	**-0.284**	0.029	**-0.241**	**-0.253**	-0.217	-0.049	-0.012	-0.140	-0.025	-0.083	0.159	-0.001
	P-value	0.055	**0.011**	**0.008**	**0.019**	0.817	**0.048**	**0.037**	0.076	0.690	0.922	0.255	0.842	0.503	0.193	0.991
Motor	*r*	-0.050	-0.073	-0.215	-0.143	0.089	-0.124	-0.011	-0.154	0.100	0.091	-0.028	0.105	-0.027	0.112	0.023
	P-value	0.690	0.562	0.086	0.256	0.480	0.326	0.934	0.221	0.429	0.473	0.825	0.405	0.830	0.371	0.856

# Remained significant after correcting for multiple comparisons.

Abbreviations: GM-CSF, Granulocyte-macrophage colony-stimulating factor; MMP-9 metalloproteinase-9; IFN-γ, interferon-γ; IL, interleukin; NGAL, neutrophil gelatinase-associated lipocalin; TNF-α, Tumor-necrosis factor-α; HIV, Human Immunodeficiency Virus; HEU, HIV-exposed uninfected.

In HEU infants (6–10 weeks) most inflammatory markers [GM-CSF (*r = 10.309, p0.022*), IFN-γ (*r = -0.339, p = 0.011*), IL-10 (*r = -0.451, p = 0.001*), IL-12p70 (*r = -0.379, p = 0.004*), IL-1β (*r = -0.491, p < 0.000*), IL-2 (*r = -0.308, p = 0.022*), IL-4 (*r = -0.418, p = 0.002*), IL-6 and NGAL (*r = -0.383, p = 0.004*)] were significantly associated with motor development in HEU children (24–28 months) after correction for multiple comparisons. MMP-9 was shown to be initially associated with motor (*r = -0.289, p = 0.034*) and language outcomes (*r = -0.305, p = 0.022*) prior to multiple comparison correction. Additionally, IL-1β (*r = -0.342, p = 0.008*) was associated with language outcomes prior to multiple comparison correction *(*Table 4*)*.

No associations of inflammatory markers in HEU children (24–28 months) with neurodevelopment measures reached statistical significance after correction for multiple comparisons. However, in HEU children (24–28 months) IL-10 (*r = -0.303, p = 0.010*) was associated with lower cognitive scores prior to multiple comparison correction. Inflammatory markers IFN-γ (*r = -0.307, p = 0.011*), IL-10 (*r = -0.319, p = 0.008*), IL-12p70 (*r = -0.284, p = 0.019*), IL-1β (*r = -0.241, p = 0.048*) and IL-2 (*r = -0.253, p = 0.037*) were also associated with language outcomes prior to multiple comparison correction *(*Table 4*)*.

There were no significant correlations between inflammatory markers in the HIV-uninfected mothers and their HU children with neurodevelopmental measures after multiple comparison corrections *(*supplementary Tables 4-6*)*.

Multivariable linear regression analyses on inflammatory markers of HEU infants at 6–10 weeks with motor development at 24–28 months showed that GM-CSF, IFN-γ, IL-10, IL-12p70, IL-1β, IL-2, IL-4, IL-6 and NGAL were associated with poorer motor development after controlling for covariates on all three models *(p < 0.05) (*Table 5*)*.

**Table 5:** Multivariable linear regression comparing inflammatory markers for HEU infants at 6–10 weeks and motor neurodevelopment scores at 24–28 months.

	Unadjusted			Model 1			Model 2			Model 3		
	B (SE)	β	P-value	B (SE)	β	P-value	B (SE)	β	P-value	B (SE)	β	P-value
GM-CSF	−2.144 (0.91)	−0.309	0.022	−2.338 (1.03)	−0.381	0.023	−2.741 (0.913)	−0.401	0.003	−2.264 (0.935)	−0.326	0.015
IFN-γ	−3.256 (1.24)	−0.339	0.011	−2.890 (1.45)	−0.310	0.047	−3.199 (1.27)	−0.337	0.012	−3.294 (1.32)	−0.351	0.013
IL-10	−5.222 (1.42)	−0.451	0.001	−5.323 (1.62)	−0.448	0.001	−5.465 (1.46)	−0.470	<0.001	−5.112 (1.53)	−0.441	0.001
IL-12p70	−3.802 (1.27)	−0.379	0.004	−3.784 (1.48)	−0.395	0.010	−4.107 (1.31)	−0.415	0.002	−3.728 (1.35)	−0.379	0.006
IL-1β	−5.810 (1.41)	−0.491	<0.001	−6.116 (1.65)	−0.522	<0.001	−5.701 (1.44)	−0.486	<0.001	−5.883 (1.56)	−0.498	<0.001
IL-2	−3.238 (1.37)	−0.308	0.022	−3.605 (1.59)	−0.353	0.023	−3.250 (1.40)	−0.314	0.021	−3.181 (1.44)	−0.310	0.027
IL-4	−3.492 (1.04)	−0.418	0.002	−3.489 (1.18)	−0.431	0.003	−3.651 (1.07)	−0.445	0.001	−3.424 (1.10)	−0.409	0.002
IL-6	−2.553 (0.99)	−0.335	0.012	−2.775 (1.20)	−0.372	0.021	−2.583 (1.034)	−0.343	0.012	−2.357 (1.12)	−0.309	0.036
NGAL	−7.460 (2.50)	−0.383	0.004	−8.655 (2.65)	−0.451	0.001	−8.563 (2.65)	−0.448	0.001	−6.659 (2.99)	−0.342	0.026

Model 1: adjusted for clinic, maternal smoking during pregnancy, maternal alcohol use during pregnancy, maternal socioeconomic status, maternal body mass index (BMI) at 6 weeks postpartum.

Model 2: adjusted for birth weight, prematurity, exclusive breastfeeding at 6–10 weeks and infant sex.

Model 3: adjusted for maternal CD4+, maternal viral load during pregnancy, maternal antiretroviral therapy (ART) regime during pregnancy and initiation of ART.

Abbreviations: GM-CSF, Granulocyte-macrophage colony-stimulating factor; IFN-γ, interferon-γ; IL, interleukin; NGAL, neutrophil gelatinase associated lipocalin.

## 4 Discussion

This is the first study reporting longitudinal associations between inflammatory markers in pregnant mothers living with HIV and uninfected pregnant mothers and their children with neurodevelopmental measures. Key findings were that maternal HIV infection was associated with decreased levels of inflammatory markers in pregnant women and in their children at 6–10 weeks and 24–28 months; there was a significant association between inflammatory markers in HEU infants at 6–10 weeks and poorer motor development at 24–28 months.

The results of this study are consistent with evidence indicating inflammatory cytokines that play an important role in immune regulation during pregnancy may be dysregulated in women living with HIV. Reduced serum levels of inflammatory cytokines in mothers living with HIV and HEU children were found, which were consistent with a few other studies. These studies found reduced levels of IL-2, IL-6 and TNF-α in mothers living with HIV and reduced levels of IL-4, IL-7 and IL-12 in HEU infants (Borges-Almeida et al., 2011, Chougnet et al., 2000, Maharaj et al., 2017). However, the current study differs from most previously reported studies, which found increased levels of inflammatory markers (predominantly IL-1β, IL-6 and TNF-α) in both mothers living with HIV and HEU children (Dirajlal-Fargo et al., 2019, Miyamoto et al., 2017, Richardson and Weinberg, 2011). The above studies were performed mostly in South America, America and European countries, which represent a population of women primarily infected with the HIV subtype Clade B; only two studies were in sub-Saharan Africa. The sub-Saharan epidemic of HIV infection, representing the greatest proportion of people living with HIV and HEU children worldwide, is largely infected with the HIV subtype Clade C (Geretti, 2006). Clade C tends to present a more immunosuppressive profile, in keeping with our findings, compared to other clades such as Clade B, which have a pro-inflammatory effect with increased neuroinflammation possibly due to the amino-acid sequence differences of the Tat protein (Rao et al., 2013, Ruiz et al., 2019). Our finding of reduced serum levels of inflammatory markers associated with HIV infection in pregnant mothers and HEU children, highlights the potential dysregulatory impact of maternal HIV infection on immune function, particularly during the early critical period of child neurodevelopment. The impact of ART on levels of inflammatory markers in mothers living with HIV and HEU children should also be considered, as this may reduce systemic inflammation and immune activation to some extent (Hileman and Funderburg, 2017). The impact of specific ART on neurodevelopmental outcomes in HEU children should also be taken into account. A study by Cassidy and colleagues in Botswana showed that exposure to efavirenz-based ART was associated with lower receptive language scores at 2 years of age as compared with HEU children with non-efavirenz-based ART (Cassidy et al., 2019). Other studies, however, have found no impact of *in utero* exposure to specific ART regimens on neurodevelopmental outcomes in HEU children at 24 months of age (Chaudhury et al., 2018, Kacanek et al., 2018) . Notably, in our analysis, ART was included as a covariate, and the inflammatory environment of the HEU children was consistently low until 2 years of age when the children were no longer exposed to ART directly. Therefore, a significant impact of ART on cytokine levels and neurodevelopmental outcomes in HEU children in this cohort appears less likely, although future research is needed to explore this area.

The reduction in cytokine serum levels in HEU children through the two years mirrors the reduced levels of cytokines found in their mothers, demonstrating a persistent effect. This suggests that the mothers’ immune profile may predict that of their children. Many studies have found that the maternal immune system significantly impacts the immune system of the fetus (Morelli et al., 2015). One potential mechanism is through the transfer of maternal cytokines across the placenta (Abu-Raya et al., 2016b, Morelli et al., 2015). Notably, the mothers living with HIV in this study had lower GM-CSF levels. GM-CSF is a cytokine that plays an important function in the survival and activation of mature myeloid cells and contributes to the maintenance of the innate immune system (Hamilton and Achuthan, 2013). Studies with GM-CSF or Csf2rb (receptor for GM-CSF) knockout mice had a reduction of conventional dendritic cells (cDC), with a most pronounced decrease in the cDC1 lineage (Bogunovic et al., 2009, Greter et al., 2012, Kingston et al., 2009). In GM-CSF deficient mice it was also found that GM-CSF is important for Th type 1 (Th1) and Th2 responses to antigenic stimulation (Wada et al., 1997). These studies indicate that insufficient GM-CSF levels lead to an impaired development of the immune system and reduced immune response. We speculate that the lower GM-CSF levels during pregnancy in women living with HIV can contribute to an altered immune development in the HEU children, which is reflected by persistent decreased pro-inflammatory (IFN-γ and IL-1β) and anti-inflammatory cytokine (IL-4) levels in the children at 6–10 weeks and 24 months of age. There are very few studies that assess the inflammatory profile of HEU children longitudinally, highlighting the importance of this study and need for future investigation in this area.

Our results demonstrated that increased levels of several immune markers (GM-CSF, IFN-γ, IL-10, IL12p70, IL-1β, IL-2, IL-4, IL-6 and NGAL) in HEU infants at 6–10 weeks were associated with poorer motor development at 2 years. This suggests that an altered immune system early in life may predict neurodevelopmental delay in later childhood. A review by Bilbo *et al* supports this by highlighting the impact of the immune system on normal brain development (Bilbo and Schwarz, 2012). HEU infants may have a more sensitive immune system compared to HU infants and consequently this may result in increased susceptibility to changes in levels of inflammatory markers. HEU infants may manifest a lower threshold for the impact of fluctuations in inflammatory markers on the developing brain. This theory is supported by evidence that HIV exposure impacts the immune system of HEU children (Abu-Raya et al., 2016a). On the other hand, the immune systems of unexposed infants are assumed to be functioning under typical physiological conditions and therefore may be able to adapt more easily to fluctuations in inflammatory markers. Previous findings in animal models suggest that maternal immune compromise also affects neurodevelopment via changes in fetal inflammatory markers (Yockey and Iwasaki, 2018).

Poorer language functioning in HEU children was associated with increased serum levels of IFN-γ, IL-10 and IL-12p70 in mothers living with HIV and their children before multiple comparison correction. This finding, though not reaching statistical significance remains important to note, as studies reporting on associations between HEU and early development have consistently found that early language outcomes are affected (Le Doare et al., 2012). We recently reported that HEU children in the DCHS (Wedderburn et al., 2019b) showed significantly lower language scores compared with HU children at 2 years of age. Further investigation into the impact of HIV exposure and mechanisms for impact on language neurodevelopment is needed.

Limitations of this study include the modest sample size warranting replication in a larger study; however, this is one of the largest studies to investigate inflammatory markers longitudinally in HEU and HU children. Although the study population is characteristic of this region of South Africa and many other parts of sub-Saharan Africa, care must be taken when extrapolating the results to other environments. The findings may not apply to populations with HIV clades other than HIV clade-C, which is the predominant clade in sub-Saharan Africa, as HIV subtypes may affect immune markers (Gandhi et al., 2009).

## 5 Conclusion

This follow-up study demonstrates that maternal HIV infection is associated with immune dysregulation with results indicating suppressed levels of serum inflammatory markers in mothers living with HIV and HEU children through to two years of age. The results further show that an altered immune system in HEU infants is associated with poorer motor development in children at two years. This study adds to the emerging understanding of the role of HIV and the immune system in neurodevelopment. Further, the findings suggest potential mechanisms through which early interventions may be developed in order to support optimal neurodevelopmental outcomes in HEU children across their lifespan.

## Declaration of Competing Interest

The authors declare that they have no known competing financial interests or personal relationships that could have appeared to influence the work reported in this paper.
