# Supplementary material for: Association of maternal and infant inflammation with neurodevelopment in HIV-exposed uninfected children in a South African birth cohort
Source: Brain Behav Immun. 2021 Jan;91:65–73. doi: 10.1016/j.bbi.2020.08.021 (PMC7772888; doi:10.1016/j.bbi.2020.08.021)
Supplement: Supplementary Data 1 [file mmc1.docx]

**Supplementary information:**

Table 1: Comparison of mean ln-transformed values of inflammatory markers between HIV-infected and HIV-uninfected mothers

|  | **Mean (SD)** | |  |  | |  |
| --- | --- | --- | --- | --- | --- | --- |
|  | **HIV-uninfected (n=190)** | **HIV-infected (n=77)** | **t** | **95% CI** | | **p-value** |
| **GM-CSF** | 4.0138 (0.90) | 3.6475 (0.85) | 3.052 | .12994 | .60256 | **.003^#^** |
| **IFN-γ** | 2.0926 (0.68) | 1.9633 (0.83) | 1.315 | -.06431 | .32308 | .190 |
| **IL-10** | 2.6231 (0.97) | 2.4113 (1.22) | 1.493 | -.06750 | .49098 | .137 |
| **IL-12p70** | 1.3389 (0.75) | 1.1674 (0.89) | 1.608 | -.03855 | .38165 | .109 |
| **IL-13** | 1.8012 (1.00) | 1.5341 (1.07) | 1.937 | -.00437 | .53864 | .054 |
| **IL-1**β | .4600 (0.70) | .2282 (0.83) | 2.316 | .03473 | .42891 | **.021** |
| **IL-2** | .8579 (0.91) | .6429 (1.09) | 1.653 | -.04117 | .47115 | .100 |
| **IL-4** | 3.4384 (1.01) | 3.0900 (1.26) | 2.370 | .05894 | .63777 | **.019** |
| **IL-5** | .9154 (0.76) | .8691 (0.86) | .432 | -.16463 | .25721 | .666 |
| **IL-6** | 1.0005 (0.96) | .9871 (1.06) | .100 | -.24988 | .27664 | .920 |
| **IL-7** | 2.4079 (0.55) | 2.3732 (0.70) | .431 | -.12398 | .19345 | .667 |
| **IL-8** | 1.6418 (0.93) | 1.6635 (0.91) | -.173 | -.26804 | .22465 | .862 |
| **TNF-α** | 1.7535 (0.54) | 1.8991 (0.62) | -1.923 | -.29474 | .00351 | .056 |
| **NGAL** | 5.1494 (0.60) | 5.0213 (0.61) | 1.566 | -.03297 | .28903 | .119 |
| **MMP-9** | 7.0693 (0.68) | 6.6142 (0.75) | 4.824 | .26937 | .64087 | **.000^#^** |

**# Remained significant after correcting for multiple comparisons.**

**Abbreviations:** GM-CSF, Granulocyte-macrophage colony-stimulating factor; IFN-γ, interferon-γ; IL, interleukin; TNF-α, tumor necrosis factor-α; NGAL, neutrophil gelatinase associated lipocalin; MMP-9, metalloproteinase-9.

Table 2: Comparison of mean ln-transformed values of inflammatory markers between HEU and HU infants at 6-10 weeks

|  | **Mean (SD)** | | | |  |  | |  | |
| --- | --- | --- | --- | --- | --- | --- | --- | --- | --- |
|  | **HU (n=155)** | | **HEU (n=61)** | | **t** | **95% CI** | | **p-value** | |
| **GM CSF** | 2.5480 | (0.98) | 2.2258 | (1.50) | 1.865 | -.01839 | .66284 | .064 |  |
| **IFN-γ** | 1.6093 | (0.92) | 1.2071 | (1.10) | 2.757 | .11462 | .68962 | **.006^#^** |  |
| **IL-10** | 2.5587 | (0.89) | 2.4162 | (0.91) | 1.057 | -.12311 | .40811 | .292 |  |
| **IL-12p70** | 0.7451 | (0.92) | 0.4567 | (1.07) | 2.000 | .00424 | .57254 | **.047** |  |
| **IL-13** | 0.9104 | (1.47) | 1.0524 | (1.57) | -.632 | -.58538 | .30129 | .528 |  |
| **IL-1**β | -0.0940 | (0.88) | -0.4650 | (0.91) | 2.771 | .10712 | .63484 | **.006^#^** |  |
| **IL-2** | 0.0810 | (0.92) | -0.1400 | (1.00) | 1.566 | -.05721 | .49921 | .119 |  |
| **IL-4** | 2.6402 | (1.22) | 2.2595 | (1.28) | 2.048 | .01432 | .74702 | **.042** |  |
| **IL-5** | 0.2544 | (0.96) | 0.3970 | (0.86) | -1.022 | -.41763 | .13244 | .308 |  |
| **IL-6** | 0.6139 | (1.20) | 0.4270 | (1.36) | .994 | -.18353 | .55732 | .321 |  |
| **IL-7** | 1.7684 | (0.72) | 1.8026 | (0.81) | -.305 | -.25545 | .18706 | .761 |  |
| **IL-8** | 2.3926 | (0.10) | 2.3708 | (1.11) | .140 | -.28357 | .32706 | .889 |  |
| **TNF-α** | 2.9360 | (0.55) | 3.0252 | (0.82) | -.928 | -.27846 | .10017 | .354 |  |
| **NGAL** | 4.6456 | (0.53) | 4.5154 | (0.58) | 1.573 | -.03293 | .29325 | .117 |  |
| **MMP-9** | 6.1367 | (0.72) | 6.0666 | (0.87) | .603 | -.15892 | .29914 | .547 |  |

**# Remained significant after correcting for multiple comparisons.**

**Abbreviations:** GM-CSF, Granulocyte-macrophage colony-stimulating factor; IFN-γ, interferon-γ; IL, interleukin; TNF-α, tumor necrosis factor-α; NGAL, neutrophil gelatinase associated lipocalin; MMP-9, metalloproteinase-9.

Table 3: Comparison of mean ln-transformed values of inflammatory markers between HEU and HU children at 24-28 months

|  | **Mean (SD)** | | | |  |  | |  |
| --- | --- | --- | --- | --- | --- | --- | --- | --- |
|  | **HU (n=190)** | | **HEU (n=76)** | | **t** | **95% CI** | | **p-value** |
| **GM CSF** | 4.7496 | (0.81) | 4.5983 | (0.95) | 1.305 | -.07696 | .37960 | .193 |
| **IFN-γ** | 2.2322 | (0.58) | 2.0086 | (0.57) | 2.851 | .06914 | .37790 | **.005^#^** |
| **IL-10** | 2.9874 | (0.66) | 2.8362 | (0.67) | 1.677 | -.02630 | .32879 | .095 |
| **IL-12p70** | 1.4775 | (0.61) | 1.2868 | (0.64) | 2.278 | .02585 | .35558 | .024 |
| **IL-13** | 2.3888 | (1.07) | 2.2004 | (0.97) | 1.336 | -.08918 | .46598 | .183 |
| **IL-1β** | 0.6314 | (0.76) | 0.2678 | (0.67) | 3.664 | .16822 | .55902 | **.000^#^** |
| **IL-2** | 0.9467 | (0.66) | 0.6757 | (0.73) | 2.924 | .08852 | .45336 | **.004^#^** |
| **IL-4** | 3.4085 | (0.93) | 3.0853 | (0.99) | 2.510 | .06961 | .57668 | **.013^#^** |
| **IL-5** | 1.3115 | (0.79) | 1.1957 | (0.70) | 1.114 | -.08879 | .32024 | .266 |
| **IL-6** | 1.2464 | (0.85) | 1.1622 | (0.90) | .717 | -.14731 | .31587 | .474 |
| **IL-7** | 2.3037 | (0.51) | 2.2253 | (0.54) | 1.108 | -.06084 | .21756 | .269 |
| **IL-8** | 2.5692 | (1.12) | 2.4684 | (1.06) | .675 | -.19329 | .39485 | .500 |
| **TNF-α** | 2.5764 | (0.66) | 2.5338 | (0.74) | .460 | -.13998 | .22527 | .646 |
| **NGAL** | 5.1152 | (0.58) | 5.0838 | (0.57) | .404 | -.12152 | .18431 | .686 |
| **MMP-9** | 6.6942 | (0.61) | 6.6613 | (0.58) | .404 | -.12731 | .19309 | .686 |

**# Remained significant after correcting for multiple comparisons.**

**Abbreviations:** GM-CSF, Granulocyte-macrophage colony-stimulating factor; IFN-γ, interferon-γ; IL, interleukin; TNF-α, tumor necrosis factor-α; NGAL, neutrophil gelatinase associated lipocalin; MMP-9, metalloproteinase-9.

Table 4: Correlation analysis between inflammatory markers in HIV-uninfected mothers with neurodevelopment measures in their children at 24-28 months

|  |  | **GM-CSF** | **IFN-γ** | **IL-10** | **IL-12p70** | **IL-13** | **IL-1β** | **IL-2** | **IL-4** | **IL-5** | **IL-6** | **IL-7** | **IL-8** | **TNF-α** | **NGAL** | **MMP-9** |
| --- | --- | --- | --- | --- | --- | --- | --- | --- | --- | --- | --- | --- | --- | --- | --- | --- |
| **Cognitive** | *r* | -.066 | -.002 | -.030 | -.051 | .048 | -.017 | -.031 | .035 | -.016 | .016 | -.072 | **.164** | .039 | .003 | .054 |
|  | p-value | .383 | .980 | .687 | .500 | .520 | .817 | .676 | .638 | .828 | .834 | .336 | **.029** | .609 | .969 | .477 |
| **Language** | *r* | -.076 | -.051 | -.020 | -.093 | .043 | -.033 | -.017 | -.004 | .063 | .002 | .011 | .123 | .014 | -.038 | -.045 |
|  | p-value | .326 | .512 | .800 | .228 | .580 | .667 | .828 | .961 | .415 | .983 | .887 | .110 | .855 | .627 | .564 |
| **Motor** | *r* | -.036 | .026 | -.004 | -.034 | .003 | .060 | .000 | .076 | .001 | -.011 | .023 | .112 | .058 | -.030 | -.014 |
|  | p-value | .635 | .727 | .955 | .650 | .968 | .432 | .996 | .315 | .986 | .888 | .764 | .138 | .441 | .693 | .853 |

**Abbreviations:** GM-CSF, Granulocyte-macrophage colony-stimulating factor; IFN-γ, interferon-γ; IL, interleukin; TNF-α, tumor necrosis factor-α; NGAL, neutrophil gelatinase associated lipocalin; MMP-9, metalloproteinase-9.

Table 5: Correlation analysis between inflammatory markers in HU infants at 6-10 weeks with neurodevelopment measures at 24-28 months

|  |  | **GM-CSF** | **IFN-γ** | **IL-10** | **IL-12p70** | **IL-13** | **IL-1β** | **IL-2** | **IL-4** | **IL-5** | **IL-6** | **IL-7** | **IL-8** | **TNF-α** | **NGAL** | **MMP-9** |
| --- | --- | --- | --- | --- | --- | --- | --- | --- | --- | --- | --- | --- | --- | --- | --- | --- |
| **Cognitive** | *r* | -.095 | -.097 | -.058 | -.127 | -.145 | **-.169** | -.120 | -.048 | -.075 | -.096 | -.080 | -.058 | -.048 | -.064 | -.067 |
|  | p-value | .253 | .243 | .485 | .128 | .081 | **.042** | .149 | .564 | .370 | .251 | .341 | .490 | .568 | .446 | .424 |
| **Language** | *r* | -.100 | -.115 | -.051 | -.165 | -.090 | **-.245** | **-.178** | -.066 | -.140 | -.150 | **-.186** | -.036 | -.069 | -.137 | **-.178** |
|  | p-value | .244 | .181 | .552 | .053 | .296 | **.004** | **.037** | .443 | .102 | .080 | **.029** | .679 | .420 | .112 | **.038** |
| **Motor** | *r* | -.044 | -.018 | -.048 | -.140 | -.095 | -.105 | -.148 | -.072 | -.131 | -.077 | -.096 | .011 | .010 | -.062 | -.117 |
|  | p-value | .600 | .829 | .569 | .093 | .257 | .209 | .075 | .393 | .117 | .358 | .252 | .896 | .904 | .462 | .164 |

**Abbreviations:** GM-CSF, Granulocyte-macrophage colony-stimulating factor; IFN-γ, interferon-γ; IL, interleukin; TNF-α, tumor necrosis factor-α; NGAL, neutrophil gelatinase associated lipocalin; MMP-9, metalloproteinase-9.

Table 6: Correlation analysis between inflammatory markers in HU children with neurodevelopment measures at 24-28 months

|  |  | **GM-CSF** | **IFN-γ** | **IL-10** | **IL-12p70** | **IL-13** | **IL-1β** | **IL-2** | **IL-4** | **IL-5** | **IL-6** | **IL-7** | **IL-8** | **TNF-α** | **NGAL** | **MMP-9** |
| --- | --- | --- | --- | --- | --- | --- | --- | --- | --- | --- | --- | --- | --- | --- | --- | --- |
| **Cognitive** | *r* | .034 | -.100 | .007 | .010 | -.071 | -.078 | -.028 | .067 | -.060 | -.106 | -.001 | -.023 | -.037 | -.030 | .034 |
|  | p-value | .651 | .184 | .929 | .892 | .347 | .299 | .708 | .376 | .427 | .157 | .990 | .759 | .625 | .688 | .654 |
| **Language** | *r* | **.162** | -.061 | -.001 | -.006 | -.101 | -.057 | -.017 | .077 | -.040 | **-.184** | -.051 | -.077 | -.089 | .083 | .054 |
|  | p-value | **.034** | .426 | .989 | .938 | .191 | .464 | .829 | .320 | .601 | **.016** | .507 | .315 | .248 | .282 | .482 |
| **Motor** | *r* | **.211** | -.055 | .025 | .067 | .015 | -.029 | -.017 | .090 | .002 | -.076 | -.003 | -.102 | -.091 | -.017 | .002 |
|  | p-value | **.005** | .470 | .746 | .378 | .846 | .699 | .819 | .235 | .980 | .315 | .965 | .179 | .230 | .819 | .977 |

**Abbreviations:** GM-CSF, Granulocyte-macrophage colony-stimulating factor; IFN-γ, interferon-γ; IL, interleukin; TNF-α, tumor necrosis factor-α; NGAL, neutrophil gelatinase associated lipocalin; MMP-9, metalloproteinase-9.
